# Editorial Notes, News and Miscellany

**Published:** 1913-10

**Authors:** 


### Editorial Notes, News and Miscellany.

---

SEE MRS. ROBBINS' ADVERTISEMENT of her Maternity Home, under the Purdue Frederick advertisement this issue.

---

Owing to the protracted illness of Dr. Duggan, editor of the Department of Eugenics, we are without his sprightly and interesting editorials this month.

---

DR. T. T. JACKSON, of San Antonio, has been appointed on the State Board of Health, vice Dr. A. W. Fly who has been appointed a member of the Board of Regents of the University of Texas.

---

FAIRCHILD BROS. & FOSTER, New York, have been awarded a gold medal for physiological pharmaceutical preparations at the exhibit in connection with the International Conference of Medicine held in London in August.

---

THE SIXTH DISTRICT (Texas) MEDICAL SOCIETY will be organized at Corpus Christi, October 13-14-15. President M. L. Graves of the Texas State Medical Association will deliver an address. Dr. Boerner will lecture on Hook Worm, and Dr. M. M. Carrick, of Dallas, will speak on Nostrums and Quackery.

---

THE FIRST NORTHER.

Now fades the glimmering straw hat out of sight,
And all the clothes a wint'ry aspect wear,
Save where the editor—always in a tight—
Must wear his linen duster still, or go bare.

Please remit.

---

SALVE FOR WOUNDED HONOR.—Dr. F. T. Griffith, a prosperous homoeopath of Austin, put a woman on the table for speculum examination. He proceeded to "make love to her" and take liberties with her person. She told her husband, who, instead of taking a stick to him, or a horsewhip, had him indicted for "assault." He was fined $50. The husband then sued him for damages for "insulting his wife." The jury awarded him $1000.

---

THE EIGHTH ANNUAL MEETING of the Medical Association of the Southwest, will be held at Kansas City, Mo., October (inst.) 7 and 8 with special clinics all day Monday, Thursday, Friday and Saturday. These clinics will embrace medicine, surgery, eye, ear, nose and throat, and genito-urinary. Headquarters, Coates Hotel, Tenth and Broadway. Dr. W. T. Wooten, Hot Springs, Ark., President; Dr. Fred H. Clark, El Reno, Okla., Secretary-treasurer.

---

THE MEDICAL ECONOMIST is the name of a sprightly little journal, the official organ of the Associated Physicians' Economic League of New York. It is edited by Dr. Thomas F. Burns and published by The Physicians' Economist Publishing Company, 71 W. 23rd street. We have received number two of volume one, September 1913, and are pleased to put the newcomer on our exchange list. Ask for a sample copy and send Dr. Burns your subscription order, $1 a year.

---

THE LAST HOPE GONE.—My small niece, aged four, came running into the room, where her mother and I were sitting.

"My dolly's sick," she said, "and I don't know what's the matter. I gave her water and she can't swallow that; the doctor gave her a pill and she can't swallow that."

"Then," said I, "I don't see but what you had better try Christian Science for her."

"We have tried it," said she, "and she can't swallow that."—*Wisconsin Medical Recorder.*

---

FOR SALE, in the suburbs of Austin, overlooking the city and the beautiful Colorado valley, a handsome two-story building, ten rooms, and ten acres of ground. This is an ideal place for a sanitarium, especially as the noted South Austin Natural Sulphur Well is on the place, together with orchard and all necessary outbuildings. In the advertising department will be found an analysis of the water. For price, terms and all details, address H. W. Williams, Austin, Texas, care of this journal, or the owner, Mrs. P. C. Beaty, 2215 S. 68th street, Philadelphia, Pa.

---

NEW OFFICERS AMERICAN FEDERATION OF SEX HYGIENE.—At a meeting of this organization which was held in Buffalo last month in connection with the Fourth International Congress of School Hygiene, the following officers were elected: President, Dr. Charles W. Eliot, president emeritus of Harvard University; vice-presidents, Dr. David Starr Jordan, of Leland Stanford University; William T. Foster, of Portland, Ore.; Felix Warburg, of New York, and W. T. Summer, of Chicago; secretary, Dr. Donald R. Hooker, of Baltimore; treasurer, Henry L. Higginson, of Boston.

It is encouraging to find the foremost educators in America leading the movement for social prophylaxis and reform.

